# Risk of Acute Lung Injury/Acute Respiratory Distress Syndrome in Critically Ill Adult Patients with Pre-Existing Diabetes: A Meta-Analysis

**DOI:** 10.1371/journal.pone.0090426

**Published:** 2014-02-27

**Authors:** Wan-Jie Gu, You-Dong Wan, Hong-Tao Tie, Quan-Cheng Kan, Tong-Wen Sun

**Affiliations:** 1 Department of Anaesthesiology, the First Affiliated Hospital, Guangxi Medical University, Nanning, China; 2 Department of Integrated Intensive Care Unit, the First Affiliated Hospital, Zhengzhou University, Zhengzhou, China; 3 The First College of Clinical Medicine, the First Affiliated Hospital, Chongqing Medical University, Chongqing, China; 4 Pharmaceutical Department, the First Affiliated Hospital, Zhengzhou University, Zhengzhou, China; Chinese Academy of Sciences, China

## Abstract

**Background:**

The impact of pre-existing diabetes on the development of acute lung injury/acute respiratory distress syndrome (ALI/ARDS) in critically ill patients remains unclear. We performed a meta-analysis of cohort studies to evaluate the risk of ALI/ARDS in critically ill patients with and without pre-existing diabetes.

**Materials and Methods:**

We searched PubMed and Embase from the inception to September 2013 for cohort studies assessing the effect of pre-existing diabetes on ALI/ARDS occurrence. Pooled odds ratio (OR) with 95% confidence interval (CI) was calculated using random- or fixed-effect models when appropriate.

**Results:**

Seven cohort **studies** with a total of 12,794 participants and 2,937 cases of pre-existing diabetes, and 2,457 cases of ALI/ARDS were included in the **meta-analysis**. A fixed-effects model meta-analysis showed that pre-existing diabetes was associated with a reduced risk of ALI/ARDS (OR 0.66; 95% CI, 0.55–0.80; *p*<0.001), with low heterogeneity among the studies (*I^2^* = 18.9%; *p* = 0.286). However, the asymmetric funnel plot and Egger's test (*p* = 0.007) suggested publication bias may exist.

**Conclusions:**

Our meta-analysis suggests that pre-existing diabetes was associated with a decreased risk of ALI/ARDS in critically ill adult patients. However, the result should be interpreted with caution because of the potential bias and confounding in the included studies.

## Introduction

Acute lung injury (ALI) is a syndrome characterized by hypoxemia, noncardiogenic pulmonary edema, low lung compliance and widespread capillary leakage. When accompanied by more severe hypoxemia (PaO2/FiO2<200 mmHg), it is called acute respiratory distress syndrome (ARDS) [1]. Development of ALI/ARDS has been associated with short and long term morbidity, prolonged hospitalization, and high health-care costs [1]. Given the clinical consequences attributable to ALI/ARDS, identifying risk factors for prevention of ALI/ARDS is of great importance and is a priority in intensive care unit (ICU).

Diabetes also is a global health priority. The prevalence of diabetes is expected to dramatically increase by the year 2025 [2]. The proportion of critically ill patients with diabetes is also growing as a result of the worldwide increase in diabetes. However, the association between diabetes and mortality among ICU patients is still debatable. In some studies, pre-existing diabetes has been a risk factor of acute kidney injury in critically ill patients [3], [4], and has been associated with an increased mortality of surgical ICU [5], but in some other studies, diabetes does not alter mortality in ICU patients [6], [7], [8].

The impact of pre-existing diabetes on the development of ALI/ARDS remains unclear in critically ill patients, especially those with one or more predisposing conditions such as sepsis/septic shock, pneumonia, trauma, and aspiration. To our knowledge, the quality and consistency of epidemiological evidence on the topic have not been systematically investigated, which is an important gap in our understanding of the effect of pre-existing diabetes on the development of ALI/ARDS. With recently accumulating evidence, therefore, we performed a meta-analysis of cohort studies to evaluate the risk of ALI/ARDS in critically ill patients with and without pre-existing diabetes.

## Materials and Methods

### Search strategy

We performed this meta-analysis in accordance with the Meta-analysis of Observational Studies in Epidemiology(MOOSE) statement [9]. PubMed and Embase databases from inception to September 2013 were searched to identify relevant studies, without language restrictions. Search terms included “diabetes”, “DM”, “acute respiratory distress syndrome”, “acute lung injury”, “ARDS”, “ALI”, and “acute respiratory failure”. In addition, we reviewed the reference lists of retrieved papers and recent reviews to identify other potentially eligible studies that we had not captured with our primary search.

### Study selection

The following inclusive selection criteria were applied: (a) study design: cohort study; (b) study population: critically ill adult patients, it meaned ICU or emergency patients with ALI/ARDS predisposing risk factors, including sepsis, septic shock, pancreatitis, pneumonia, aspiration, trauma, or high-risk surgery; (c) comparison intervention: with and without pre-existing diabetes; and (d) outcome measure: the development of ALI/ARDS. In the case of duplicate data publication (several studies with overlapping samples), we only included the most informative article or complete study to avoid duplication of information.

### Data extraction and quality assessment

A standardized data collection form was used to extract the following information from each included article: first author, publication year, sample size, number of diabetes cases, number of ALI/ARDS cases, population characteristics, type of study design, definition of ALI/ARDS, and odds ratios (ORs) with the corresponding 95% confidence intervals (CIs) on multivariable analysis. The supplementary files were also examined for data extraction. Where necessary, we contacted authors of included studies for additional information.

We assessed the methodological quality of each study on 8 items used in the Newcastle-Ottawa Scales (NOS) [10]. We assigned risk of bias categories based on the number of Newcastle–Ottawa Scales (NOS) items judged inadequate in each study, as follows: low risk of bias (0–1 inadequate item); medium risk of bias (2–3 inadequate items); high risk of bias (over 3 inadequate items); very high risk of bias (no description of methods).

Two investigators (WJ Gu and YD Wan) independently conducted the study selection, data extraction and quality assessment, a third investigator (TW Sun) was consulted to resolve any discrepancies.

### Statistical analysis

OR was used as a common measure of the association between pre-existing diabetes and the development of ALI/ARDS across studies. Heterogeneity was tested using the Cochran Q statistic (*p*<0.1) and quantified with the I^2^ statistic, which describes the variation of effect size that is attributable to heterogeneity across studies [11]–[13]. An *I^2^* value greater than 50% indicates significant heterogeneity. The value of the *I^2^* statistic was used to select the appropriate pooling method: fixed-effects models were used for *I^2^*< 50% and random-effects models for *I^2^*≥50% [11]–[13]. To explore the possible source of heterogeneity and to examine the influence of various clinical factors on the overall risk estimate, we further carried out *prior* subgroup analyses according to study design (prospective vs. retrospective), predisposing conditions (sepsis/septic shock vs. >1 predisposing conditions), study center (single-center vs. multicenter), and sample size(≤1000 vs. >1000). We also investigated the influence of a single study on the overall pooled estimate by omitting one study in each turn. Potential publication bias was detected by Begg's funnel plots and Egger's regression test [14], [15]. A *p* value <0.05 was judged as statistically significant, except where otherwise specified. All statistical analyses were performed using STATA, version 11.0 (Stata Corp).

## Results

### Study selection and study characteristics

**Figure 1:**
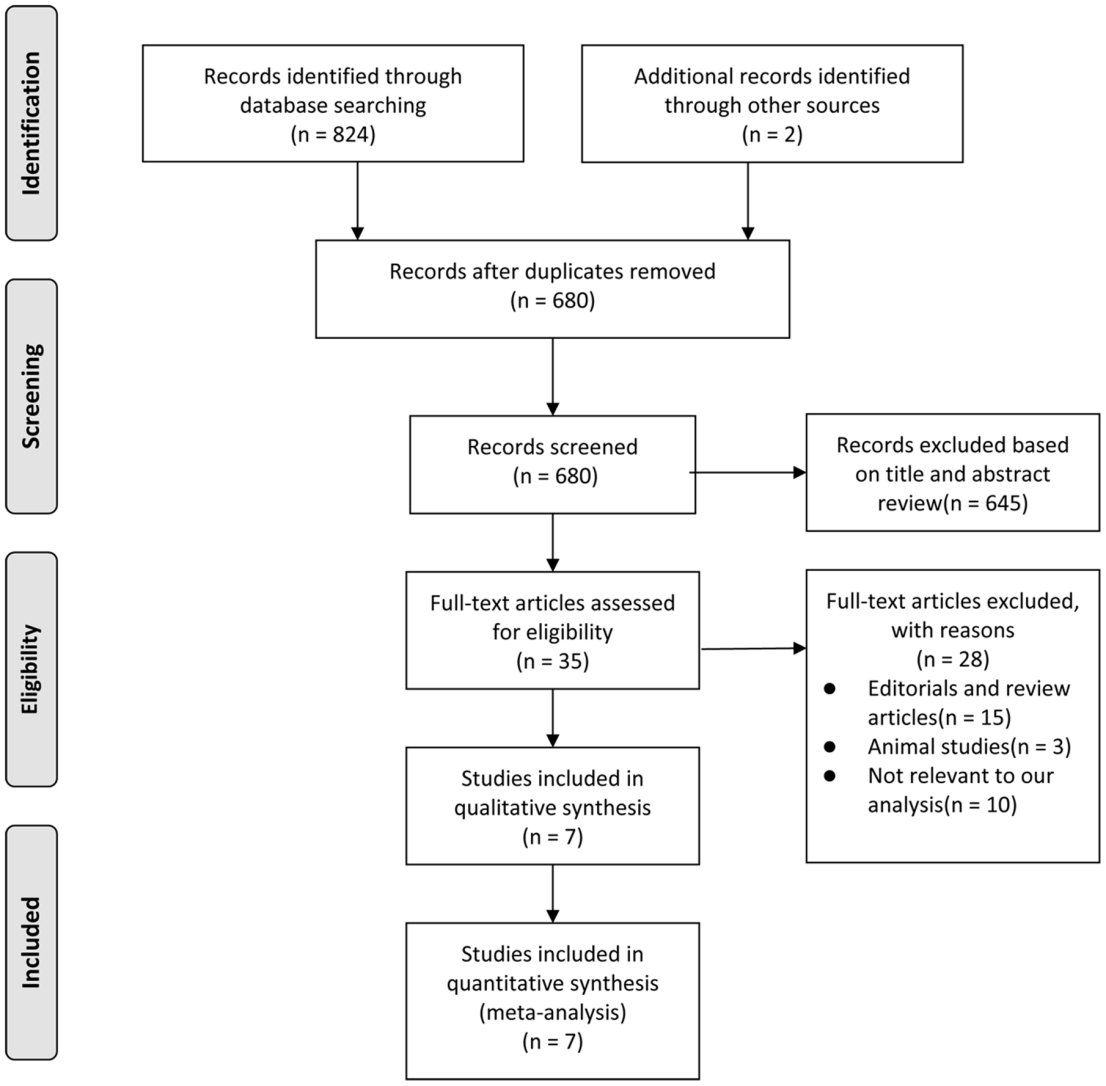
Flow diagram for selection of articles.

The initial search yielded 826 relevant publications, of which 819 were excluded for duplicate studies and various reasons (reviews, animal studies, or not relevant to our analysis) on the basis of the title/abstract and full text (see the detail in Figure 1). The remaining seven cohort studies were included in the final analysis [15]–[21].

The main characteristics of the seven included cohort studies are shown in Table 1. These studies were published between 2000 and 2013. In total, 12,794 participants, 2,937 cases of pre-existing diabetes, and 2,457 cases of ALI/ARDS were enrolled. All the included studies use the same definition of ALI/ARDS (American-European Consensus Conference definition) [22]. The average NOS score of the studies included was 6.7 (range from 6 to 7). An additional file shows this in more detail [see File S1].

**Table 1:** Main characteristics of cohort studies included in the meta-analysis.

Author/Year	Study size	Number of diabetes	Number of ALI/ ARDS	Population	Study design	Definition of ALI/ ARDS	OR (95% CI) on multivariate analysis	Adjustment for Covariates
Moss, 2000 [15]	113	32	46	Adult ICU patients with septic shock	PC	American-European Consensus Conference definition	0.33 (0.12–0.90)	Age, source of infection, and history of cirrhosis
Gong, 2005 [16]	688	164	221	Adult ICU patients with>1 predisposing ARDS condition	PC	American-European Consensus Conference definition	0.58 (0.36–0.92)	Predisposing ARDS conditions: Sepsis syndrome, septic shock, direct pulmonary injury; Other: Age, APACHE III, transfusion of PRBCs, number of PRBCs transfused, and hematologic failure
Iscimen, 2008 [17]	160	55	71	Adult ICU patients with septic shock	PC	American-European Consensus Conference definition	0.44 (0.17–1.07)	Predisposing ARDS conditions: Aspiration; Other: Age, APACHE III, alcohol abuse, chemotherapy, delayed goal-directed resuscitation, delayed antibiotics, tachypnea, transfusion of PRBCs
Gajic, 2011 [18]	5584	1295	377	Adult hospitalized patients with sepsis	PC	American-European Consensus Conference definition	0.55 (0.25–1.16)	Predisposing ARDS conditions: Aspiration, high-risk surgery, high-risk trauma, pancreatitis, pneumonia, shock Other: Sex, APACHE II, admission source, acidosis, alcohol abuse, chemotherapy, emergency surgery, hypoalbuminemia, obesity, oxygen supplementation, smoking, tachypnea
Trillo-Alvarez, 2011 [19]	409	87	68	Adult ICU patients with>1 predisposing ARDS condition	RC	American-European Consensus Conference definition	0.16 (0.03–0.77)	Predisposing ARDS conditions: Aspiration, emergent high-risk surgery, sepsis, shock; Other: Alcohol abuse, hypoalbuminemia, oxygen supplementation, smoking, tachypnea
Koh, 2012 [20]	2013	317	720	Adult ICU patients >1 predisposing ARDS condition	RC	American-European Consensus Conference definition	0.76 (0.43–1.33)	Age, sex, BMI, myocardial infarction, medication
Yu, 2013 [21]	3827	987	954	Adult ICU patients with >1 predisposing ARDS condition	PC	American-European Consensus Conference definition	0.75 (0.59–0.94)	Predisposing ARDS conditions: Multiple transfusion, septic shock, trauma Other: Age, APACHE III, BMI, ICU admission hyperglycemia, renal failure, hematologic failure, alcohol use, smoking, medication

*ALI/ARDS acute lung injury/acute respiratory distress syndrome, APACHE Acute Physiology and Chronic Health Evaluation, BMI body mass index, ICU intensive care unit, PC prospective cohort, PRBCs packed red blood cells, RC retrospective cohort.*

### Pre-existing diabetes and the risk of ALI/ARDS

**Figure 2:**
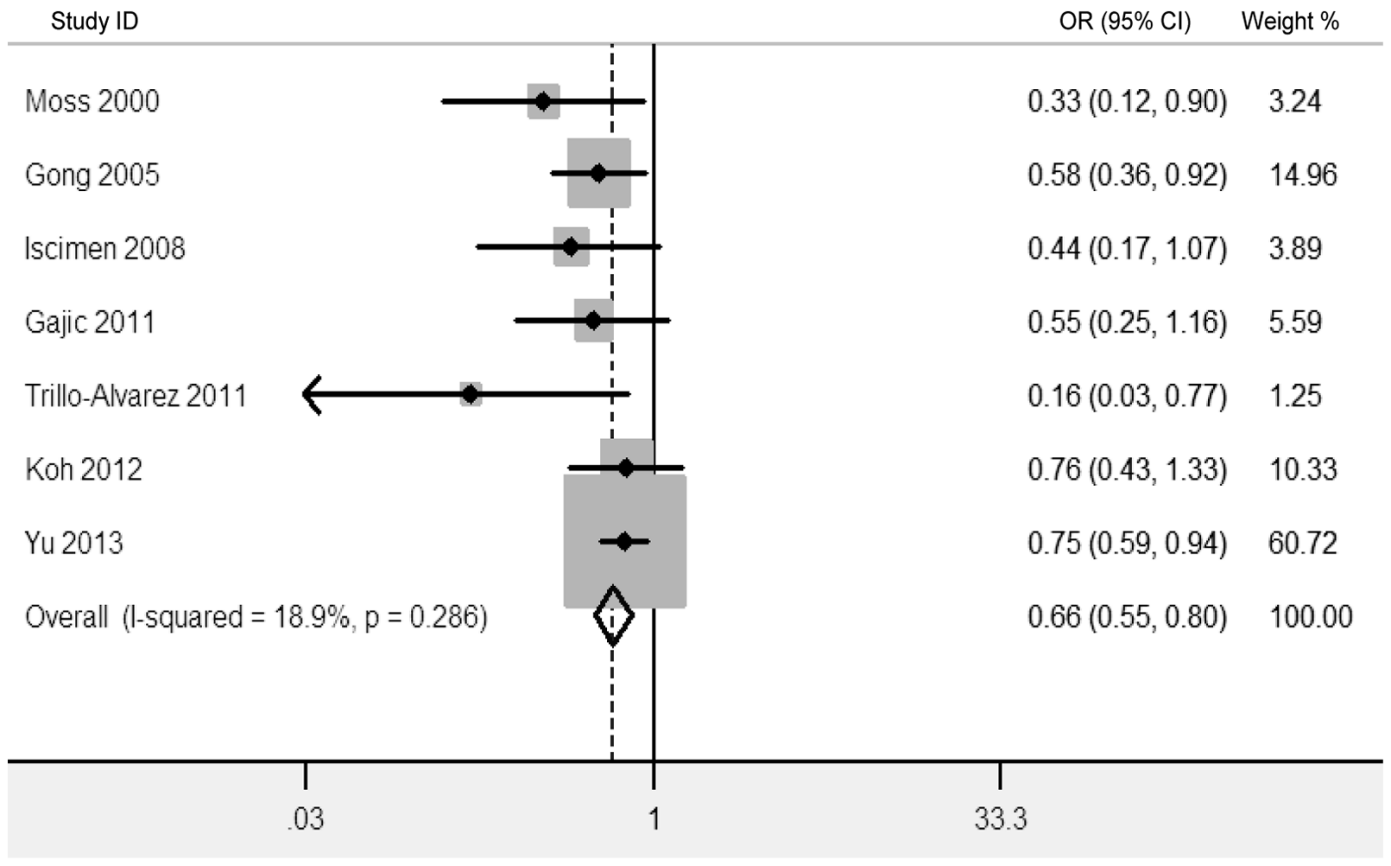
Forest plot showing the risk of ALI/ARDS in critically ill adult patients with pre-existing diabetes.

Figure 2 shows the pooled results from the fixed-effects model combing the ORs for ALI/ARDS. Overall, 12,794 patients were included in this analysis (2,937 cases of pre-existing diabetes and 2,457 cases of ALI/ARDS). Pre-existing diabetes was associated with a decreased risk of ALI/ARDS in critically ill adult patients (OR 0.66; 95% CI, 0.55–0.80; *p*<0.001), with low heterogeneity among the studies (*I^2^* = 18.9%; *p* = 0.286). Further exclusion of any single study did not materially alter the overall combined OR, with a range of from 0.55 (95% CI, 0.41–0.74) to 0.68 (95% CI, 0.56–0.83). Table 2 shows the results of subgroup analyses for ALI/ARDS. The finding of decreased risk of ALI/ARDS in critically ill adult patients was consistently found in most subgroup analyses.

**Table 2:** Subgroup analyses for ALI/ARDS.

Subgroup	No. of studies	OR (95% CI)	*p*_heterogeneity_	*I^2^*
Total [15]–[21]	7	0.66 (0.55–0.80)	0.286	18.9%
Study design				
Prospective cohort [15]–[18], [21]	5	0.67 (0.55–0.81)	0.377	5.2%
Retrospective cohort [19], [20]	2	0.64 (0.38–1.09)	0.075	68.4%
Predisposing conditions				
Sepsis/septic shock [15], [17], [18], [31]	4	0.70 (0.57–0.87)	0.246	27.6%
>1 predisposing conditions [16,21–21]	4	0.70 (0.58–0.85)	0.239	28.8%
Study center				
Single-center [16], [17], [19], [20]	4	0.58 (0.42–0.81)	0.302	17.7%
Multicenter [15], [18], [31]	3	0.70 (0.57–0.88)	0.240	29.9%
Sample size				
≤1000 [15]–[17], [19]	4	0.48 (0.33–0.70)	0.399	0%
>1000 [18], [20], [31]	3	0.73 (0.60–0.90)	0.744	0%

*ALI/ARDS acute lung injury/acute respiratory distress syndrome.*

### Publication bias

**Figure 3:**
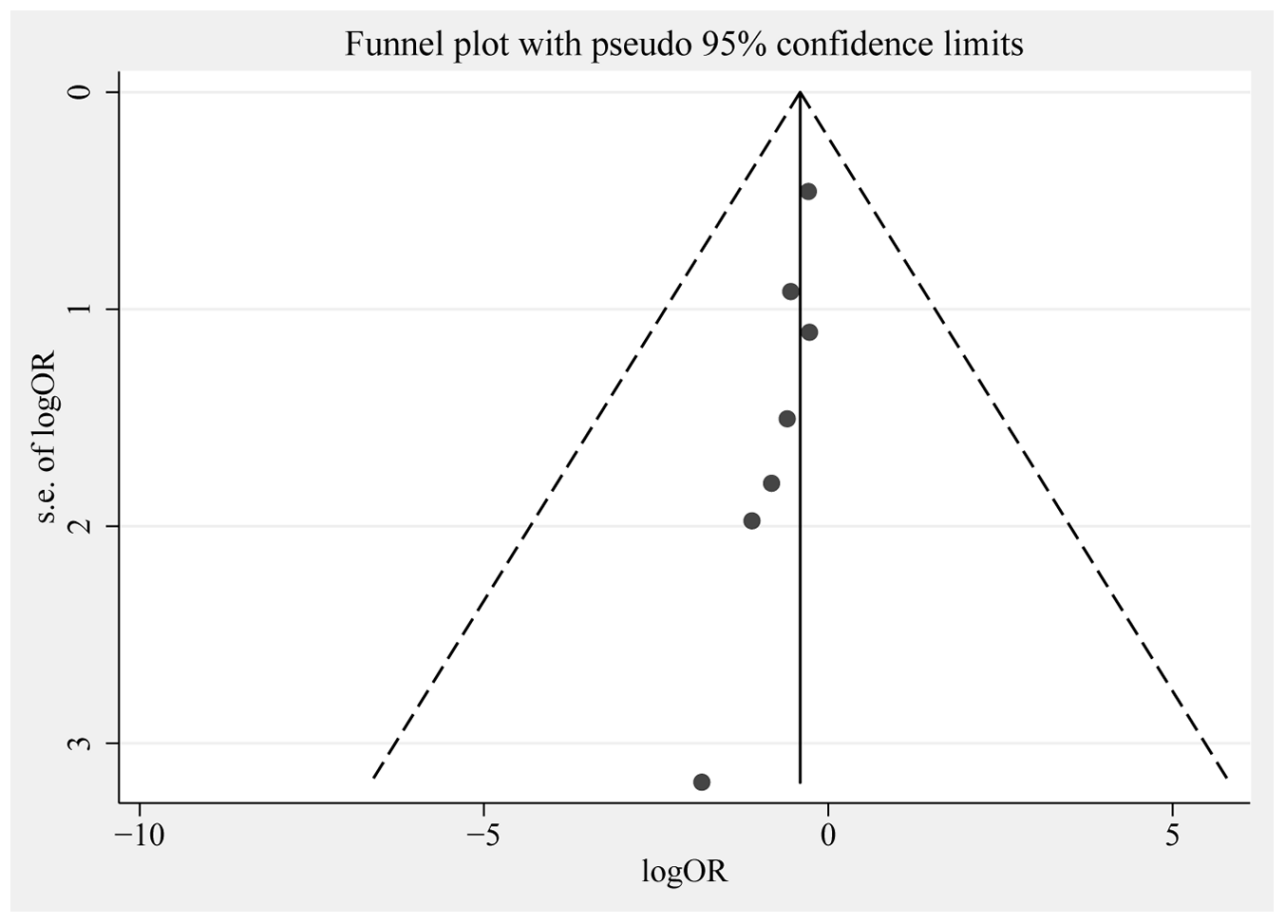
Funnel plot for the risk of ALI/ARDS in critically ill adult patients with pre-existing diabetes.or, risk ratio. s.e., standard error.

Egger's test (*p* = 0.007) suggested that publication bias may exist. Also, there was asymmetry in the lower segments of the funnel plot in which small negative trials were missing (Figure 3), potentially leading to overstatement of the treatment effect. But the low power with only seven studies limited the interpretability of the finding.

## Discussion

### Main findings

To the best of our knowledge, this is the first meta-analysis to explore the association between pre-existing diabetes and the risk of ALI/ARDS in critically ill adult patients. Our meta-analysis of seven cohort studies suggests that pre-existing diabetes was associated with a decreased risk of ALI/ARDS in critically ill adult patients. In addition, the association was consistently found in most subgroup analyses.

### Possible mechanism

Although pre-existing diabetes may decrease the risk of ALI/ARDS, the mechanism remains unclear. Since inflammation plays an important role in the onset and progression of ALI/ARDS [23], several studies have suggested that the protective effect of diabetes for ALI/ARDS may result from the reduced inflammatory response [24], [25]. Filgueiras and colleagues [24] reported that in non-diabetic rats with ALI/ARDS secondarily to sepsis, the lung presented edema, leukocyte infiltration, and increased COX2 expression; however, these inflammatory events were less intense in diabetic rats. Similarly, in the diabetic mice with infection, the inflammatory response is lower compared with non-diabetic mice [25]. Decreased inflammatory response may be associated with attenuation of cytokine release and reduction of neutrophil migration [25]. The involved cytokines included PPAR-γ [26], nuclear factor-κB [27], insulin-like growth factor-1 [27], [28], leptin, and development of advanced glycation end products [29], [30]. For the neutrophil migration, α1-acid glycoprotein is important in the failure of this process after sepsis [31], but the mechanism is not completely understood.

On another note, diabetes therapy modifies some effects that diabetes may have on ALI/ARDS. Insulin is usually the only diabetic treatment that is continued during critical illness. Independent of glycemic control, insulin has been shown to be immunomodulatory Independent of glycemic control [32]. In clinical studies, intensive insulin therapy may decrease mortality for critically ill patients [33], However, it is difficult to know whether insulin may be detrimental or beneficial in ALI/ARDS. According to our study, diabetes is protective in ALI/ARDS, then reversal of the anti-inflammatory effects of diabetes by insulin may negate the beneficial effect of diabetes on ALI/ARDS. Alternatively, if insulin reverses the chronic immunosuppressive effects of diabetes and restores the balance in inflammatory response, then insulin may be potentially beneficial [34]. More studies are needed to determine whether insulin is detrimental or beneficial in ALI/ARDS. Other drug commonly used was metformin, which was recently shown to reduce oxidative injury [35]. But the possible mechanism of diabetes therapy on ALI/ARDS still remains unclear.

### Clinical implications

Our findings are of clinical significance to some extent. The most consistent evidence was the decreased risk of ALI/ARDS in critically ill adult patients with pre-existing diabetes. Currently, physicians caring for critically ill patients can not accurately determine which patient will develop ALI/ARDS, since no biological markers have been found to predict it precisely so far [36], [37]; however, physicians could roughly predict who was more likely to worsen to ALI/ARDS according to the risk factors in critically ill patients. The following risk factors have been found: alcohol abuse [38], hypoalbuminemia [15], [39], transfusions [15], tachypnea [16], high tidal volumes [40], and obesity [41]. Besides, diabetes, as another risk factor of ALI/ARDS has been paid more attention [14]–[20]. However, to our knowledge, whether pre-existing diabetes will protect patients from converting to ALI/ARDS or facilitate the development of ALI/ARDS remains still unknown. Our meta-analysis suggested pre-existing diabetes was associated with a decreased risk of ALI/ARDS in critically ill adult patients. The findings, for the clinicians, may contribute to the sorting and management of critically ill patients with diabetes, as a protective factor for ALI/ARDS. As a primary prevention strategy, excluding risk factors is equally important to identifying those. Diabetes may be excluded from the high risk factors of ALI/ARDS.

### Strengths and limitations

Strengths of this meta-analysis included its exhaustive search without language restrictions and validated systematic review methods following the MOOSE guidelines. Also, all included studies were cohort studies and high-quality, with an average NOS score of 6.7 allowing for the impossibility of randomized controlled trial. High-quality cohort studies may minimize the likelihood of recall bias, which is of particular concern in other epidemiological study designs.

Several limitations of this meta-analysis merit consideration. First, the characteristics of populations, the type of diabetes (type 1 or type 2), the definition of diabetes, severity of the illness, and the adjusted confounding factor were not strictly described in some trials. These factors may result in heterogeneity and have a potential impact on our results. Second, the critically ill patients was really a broad population, in our study, most studies described it as ICU patients with one or more ALI/ARDS predisposing risk factors, including sepsis, septic shock, pancreatitis, pneumonia, aspiration, trauma, or high-risk surgery, etc. So whether the conclusion was suitable for the unselected ICU population remain questionable. Third, both the asymmetric funnel plot and Egger's test suggested publication bias may exist. Small negative trials were missing, potentially resulting in overstatement of the treatment effect. Furthermore, we are unable to assess the impact of pre-existing diabetes on other clinically meaningful end points, such as mortality attributed to ALI/ARDS, because of sparse and inconsistent reporting across studies.

## Conclusions

In summary, this meta-analysis of seven cohort studies suggests that pre-existing diabetes decreased the risk of ALI/ARDS in critically ill adult patients; however, the result should be interpreted with caution because of the potential bias and confounding in the included studies.

## Supporting Information

Checklist S1 **PRISMA Checklist.**
(DOC)

File S1 **Methodological quality assessment (risk of bias) of included studies by Newcastle-Ottawa Scales.**
(DOC)
