# Supplementary material for: Risk of Acute Lung Injury/Acute Respiratory Distress Syndrome in Critically Ill Adult Patients with Pre-Existing Diabetes: A Meta-Analysis
Source: PLoS One. 2014 Feb 27;9(2):e90426. doi: 10.1371/journal.pone.0090426 (PMC3937384; doi:10.1371/journal.pone.0090426)
Supplement: File S1 — Methodological quality assessment (risk of bias) of included studies by Newcastle-Ottawa Scales. (DOC) [file pone.0090426.s002.doc]

| **Study** | **Selection** | | | | **Comparability** | **Outcome** | | | **Total score** |
| --- | --- | --- | --- | --- | --- | --- | --- | --- | --- |
|  | Exposed Cohort | Nonexposed Cohort | Ascertainment of exposure | Outcome of interest |  | Assessment of outcome | Length of follow-up | Adequacy of follow-up |  |
| Moss, 2000 [11] | * | * | * | * | ** | * | - | - | 7 |
| Gong, 2005 [12] | * | * | * | * | ** | * | - | - | 7 |
| Iscimen, 2008 [13] | * | * | * | * | ** | * | - | - | 7 |
| Gajic, 2011 [14] | * | * | * | * | ** | * | - | - | 7 |
| Trillo-Alvarez, 2011 [15] | * | * | * | - | ** | * | - | - | 6 |
| Koh, 2012 [16] | * | * | * | - | ** | * | - | - | 6 |
| Yu, 2013 [17] | * | * | * | * | ** | * | - | - | 7 |

**Additional file 1** Methodological quality assessment (risk of bias) of included studies by Newcastle-Ottawa Scales
